# Adenovirus Vector-Derived VA-RNA-Mediated Innate Immune Responses

**DOI:** 10.3390/pharmaceutics3030338

**Published:** 2011-07-11

**Authors:** Mitsuhiro Machitani, Tomoko Yamaguchi, Kahori Shimizu, Fuminori Sakurai, Kazufumi Katayama, Kenji Kawabata, Hiroyuki Mizuguchi

**Affiliations:** 1 Laboratory of Biochemistry and Molecular Biology, Graduate School of Pharmaceutical Sciences, Osaka University, 1-6, Yamadaoka, Suita, Osaka, 565-0871, Japan; 2 Laboratory of Stem Cell Regulation, National Institute of Biomedical Innovation (NiBio), Osaka,567-0085, Japan; 3 Laboratory of Biomedical Innovation, Graduate School of Pharmaceutical Sciences, Osaka University, 1-6, Yamadaoka, Suita, Osaka, 565-0871, Japan; 4 The Center for Advanced Medical Engineering and Informatics, Osaka University, 1-6, Yamadaoka, Suita, Osaka, 565-0871, Japan

**Keywords:** adenovirus vector, VA-RNA, gene therapy, innate immunity

## Abstract

The major limitation of the clinical use of replication-incompetent adenovirus (Ad) vectors is the interference by innate immune responses, including induction of inflammatory cytokines and interferons (IFN), following *in vivo* application of Ad vectors. Ad vector-induced production of inflammatory cytokines and IFNs also results in severe organ damage and efficient induction of acquired immune responses against Ad proteins and transgene products. Ad vector-induced innate immune responses are triggered by the recognition of Ad components by pattern recognition receptors (PRRs). In order to reduce the side effects by Ad vector-induced innate immune responses and to develop safer Ad vectors, it is crucial to clarify which PRRs and which Ad components are involved in Ad vector-induced innate immune responses. Our group previously demonstrated that myeloid differentiating factor 88 (MyD88) and toll-like receptor 9 (TLR9) play crucial roles in the Ad vector-induced inflammatory cytokine production in mouse bone marrow-derived dendritic cells. Furthermore, our group recently found that virus associated-RNAs (VA-RNAs), which are about 160 nucleotide-long non-coding small RNAs encoded in the Ad genome, are involved in IFN production through the IFN-β promoter stimulator-1 (IPS-1)-mediated signaling pathway following Ad vector transduction. The aim of this review is to highlight the Ad vector-induced innate immune responses following transduction, especially VA-RNA-mediated innate immune responses. Our findings on the mechanism of Ad vector-induced innate immune responses should make an important contribution to the development of safer Ad vectors, such as an Ad vector lacking expression of VA-RNAs.

## 1. Introduction

The first-generation E1-deleted adenovirus (Ad) vector (FG-Ad vector) is one of the most promising vectors for gene therapy, as well as basic research due to its advantages as a gene delivery vehicle. FG-Ad vectors are relatively easy to construct, can be produced at a high titer, and have a high transduction efficiency into a wide spectrum of dividing and non-dividing cells *in vitro* and *in vivo*. However, application of FG-Ad vectors has been limited to local administration in most clinical trials of gene therapy, since the systemic administration of Ad vectors induces both adaptive and innate immune responses with its humoral and cell-mediated components [1,2].

In the case of adaptive immune response, capsid antigens are largely responsible for the specific immunity toward Ad vectors. In addition, in the FG-Ad vector, leaky expression of viral genes from the vector stimulates an immune response against Ad vector-transduced cells [3,4]. In a replication-incompetent FG-Ad vector, the E1A gene, which encodes an essential transactivator of the other viral genes, is deleted, in addition to the E1B gene. Therefore, theoretically, no other viral genes should be expressed following transduction; however, viral genes from the vector genome, including the E2A and E4 genes, are indeed expressed, which leads to an induction of cellular immunity against Ad proteins as well as to Ad protein-induced toxicity [5-11]. Such Ad protein-induced cellular immunity and toxicity frequently cause both an elimination of Ad vector-transduced cells and tissue damages, leading to short-lived transgene expression [5-7,9,10,12,13]. We recently quantitatively analyzed the leaky expression profiles of Ad genes using real-time RT-PCR following transduction with FG-Ad vectors [14]. The pIX and E4 genes were most highly expressed among the Ad genes examined, and their expression levels were 1/50 to 1/5000 of the expression levels observed with the same MOI of the wild-type Ad. The cytotoxic T lymphocyte (CTL) response can be elicited against these viral gene products and/or transgene products expressed in the transduced cells. On the other hand, the expression levels of E2B and major capsid proteins (including the hexon, penton base, and fiber proteins) were almost the same or even slightly above those observed in mock-transduced cells [14].

To reduce cell-mediated immune responses against viral gene products expressed in the transduced cells, “helper-dependent (HD)” or “gutted” Ad vectors have been developed. In this vector, all viral genes are deleted except the inverted terminal repeat (ITR) sequences at both ends and the packaging signal. The deletion of all viral protein-coding regions from the Ad genome improves the prospects of Ad vectors for long-term gene expression, suggesting that the immunogenic toxicities induced by HD-Ad vectors are greatly reduced [15].

Humoral virus-neutralizing antibody responses against the Ad capsid itself are another limitation, preventing transduction upon the subsequent administration of vectors of the same serotype. Because hexons are primarily targeted by neutralizing antibodies, hexon modification has been reported to allow for escape from neutralizing antibodies [16]. As other strategies, Ad vectors belonging to subgroups other than Ad serotype 5, such as Ad serotype 11 or 35, or to species other than humans, have also been developed [17-21].

Compared with the adaptive immunity to Ad vectors, the mechanism of Ad vector-induced innate immune responses is less understood; however, several studies have reported which cellular molecules are involved in and which components of Ad trigger the Ad vector-induced innate immune responses [22-25]. In addition, we demonstrated that virus associated RNA (VA-RNA) I and II, which are about 160 nucleotide-long non-coding RNAs encoded in the Ad genome (bp 10620-10779 and 10876-11038), are transcribed from a conventional FG-Ad vector as well as the wild-type Ad, and that VA-RNAs trigger innate immune responses through the IFN-β promoter stimulator-1 (IPS-1) [23]. In this report, we review the Ad vector-mediated innate immune responses, especially VA-RNA-mediated innate immune responses.

## 2. Ad vector-Mediated Innate Immune Responses

### 2.1. Biodistribution of intravenously injected Ad vectors and production of cytokines/chemokines

Systemically administered Ad vectors are rapidly cleared from the blood of mice, with a half-life of less than 3 min [26-28]. Liver Kupffer cells play a central role in clearing the Ad genome from the bloodstream [29-31]. It has been proposed that a low dose of Ad vectors (∼10^10^ vector particles) is rapidly sequestered by Kupffer cells (liver macrophages), while higher doses of Ad vectors are delivered into both Kupffer cells and hepatocytes, leading to a nonlinear dose response in hepatic transgene expression [32]. At a dose of 3.0 × 10^10^ vector particles per mouse, Ad vectors are likely to be equally distributed to the Kupffer and hepatocytes [27,33]. Despite the high uptake of Ad vectors by Kupffer cells, the Ad vector-mediated transduction efficiencies in Kupffer cells were much lower than those in the hepatocytes, indicating the uptake of Ad vectors by Kupffer cells is a function of phagocytosis rather than a receptor-mediated infectious pathway [28]. The spleen is the second organ that intravenously injected Ad vectors accumulate in. Since liver and spleen contain many immune cells, including liver Kupffer cells, splenic dendritic cells (DCs) and macrophages, these cells had been assumed to be responsible for the production of inflammatory cytokines/chemokines such as interleukin (IL)-6, tumor necrosis factor (TNF)-α, and Interferon gamma-induced protein 10 kDa (IP-10), and regulated on activation, normal T cell expressed and secreted (RANTES). The production of these inflammatory cytokines/chemokines causes the activation of an innate immune response [34,35]. Recent reports suggested that the spleen is the major site of cytokine, chemokine, and interferon (IFN) production. When the mice are splenectomized, IL-6 production is decreased upon Ad vector injection [36]. Reverse transcriptase-polymerase chain reaction (RT-PCR) analysis of the liver and spleen after systemic Ad vector injection suggests that IL-6 as well as other immune cytokines, chemokines, and IFNs were mainly produced from the spleen, especially from conventional dendritic cells (DC) (CD11c^+^B220^−^ cells), not the liver [37]. The fiber-modified Ad vector containing a stretch of lysine residues (K7 (KKKKKKK) peptide), which target heparan sulfates on the cellular surface, greatly reduced IL-6 production after intravenous injection into mice, possibly due to the reduced biodistribution of the Ad vector to the spleen [37].

Excessive complement activation has also been reported to be involved in Ad vector-mediated innate immune responses [38,39]. Furthermore, Xu *et al.* reported that Ad vectors are opsonized by immunoglobulin M antibodies and complement, leading to clearance of Ad vectors by Kupffer cells, which is dependent on scavenger receptors [40]. Blood coagulation factor X binds to hypervariable regions of the Ad5 hexon, leading to liver transduction and hepatotoxicity [41-45]. Modification of the hypervariable regions of hexon resulted in decreases of Ad vector-transduction of hepatocytes with potential evasion from Kupffer cells [46]. The interaction of Ad vectors with blood factors and the subsequent uptake by Kupffer cells might also play a role in the induction of innate immune responses.

### 2.2. Signaling pathway leading to innate immune responses

The possible components of FG-Ad vectors responsible for activating innate immune responses are capsid proteins, the viral genome (DNA), and viral transcripts. Induction of the antiviral innate immune response depends on the recognition of viral components by host pattern recognition receptors (PRRs). The most well-known PRRs are the Toll-like receptors (TLRs) that recognize pathogen-associated molecular patterns (PAMPs), including lipopolysaccharides, double-stranded RNA (dsRNA), single-strand RNA (ssRNA), and unmethylated CpG DNA [47]. After the recognition of PAMPs by TLRs, all of the TLRs, with the exception of TLR3, transduce intracellular signaling through the adaptor protein myeloid differentiating factor 88 (MyD88), which initiates a signaling cascade leading to the activation of NF-κB and IFN regulatory factors (IRFs). We and others have reported that Ad vectors elicit the production of inflammatory cytokines such as IL-6 and IL-12 in a TLR9-dependent manner in conventional dendritic cells (cDCs) [24,48]. Zhu *et al.* [49] demonstrated that the Ad vector-induced production of type I IFN by cDCs is mediated by a TLR9-independent pathway, whereas the Ad vector-induced production of type I IFN by plasmacytoid DCs is mediated by a TLR9-dependent pathway. The signaling pathway for type I IFN production after Ad vector treatment is known to differ from that for inflammatory cytokine production. In another PRR pathway, cytosolic RNAs are recognized by retinoic acid-inducible gene I (RIG-I)-like receptors, which include RIG-I and melanoma differentiation-associated gene 5 (Mda5) [50-52]. RIG-I and Mda5 contain RNA helicase domains that recognize viral dsRNA [53,54]. In addition, RIG-I recognizes ssRNA containing 5′-triphosphate [55-57]. RIG-I and Mda5 also contain N-terminal tandem caspase activation and recruitment domains (CARDs), which interact with the CARD domain of IFN-β promoter stimulator-1 (IPS-1, also known as MAVS, VISA, and Cardif) [58]. This interaction finally activates several transcriptional factors (e.g., NF-κB, IRF3, and IRF7) and induces the production of inflammatory cytokines and type I IFN.

## 3. VA-RNA-Mediated Innate Immune Response and an Ad Vector Lacking the Expression of VA-RNAs

### 3.1. Function of VA-RNAs and VA-RNA-mediated innate immune response

**Figure 1.**
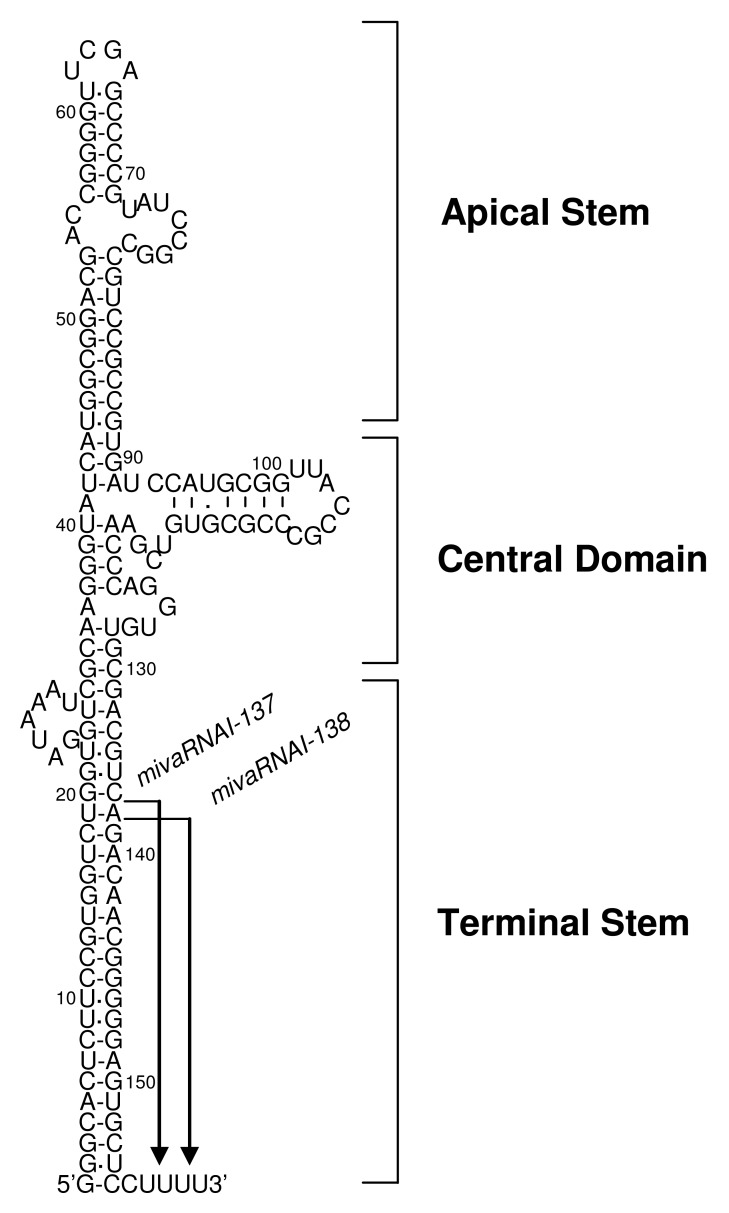
Secondary structure of VA-RNA-I. VA-RNA is composed of the apical stem, the central domain, and the terminal stem. VA-RNA-I is processed into two major species of mivaRNAs (mivaRNAI-137 and 138).

The Ad genome encodes two non-coding small RNAs, VA-RNA I (a major species) and VA-RNA II (a minor species), which are about 160 nucleotide-long non-coding RNAs encoded in the Ad serotype 5 genome regions, respectively, and which are transcribed by RNA polymerase III [59]. VA-RNAs have two internal transcription control elements (*box A* and *box B*), which in the case of VA-RNA I derived from Ad serotype 5 are located at bp 10629-10637 and bp 10673-10688, respectively [60,61]. Sequences of VA-RNAs vary considerably among Ad vectors from different serotypes; however, structural characteristics (*i.e.*, the apical stem, the central domain, and the terminal domain) are conserved in all VA-RNAs and are structurally highly stable (Figure 1) [62,63]. The apical stem and the central domain are involved in Ad amplification through the association with double-stranded RNA-dependent protein kinase (PKR), as described below. The apical stem is essential for the association with PKR [64-67] and the central domain is required for efficient inhibition of PKR activation [68-71]. The terminal stem is recognized by Exportin-5, which is essential for nuclear export of VA-RNAs [72]. Furthermore, the terminal stem may be required for the stabilization of other domains and the protection from exonuclease activity *in vivo* [63]. VA-RNA I is rapidly synthesized and accumulated to very high levels, 10^8^ molecules per cell, while VA-RNA II is synthesized at 20-fold lower levels, 5 × 10^6^ molecules per cell, during the late phase of infection [59]. VA-RNAs are transcribed from a conventional FG-Ad vector as well as the wild-type Ad, and this transcription depends on RNA polymerase III [23].

In previous studies, VA-RNA I-deleted Ad grew 10-fold less well, while VA-RNA II-deleted Ad grew as well as the wild-type Ad; however, both VA-RNA I- and II-deleted Ad grew 60-fold less well (note that these VA-RNA mutant Ads contain the E1 gene) [73-77]. These results suggest that VA-RNA I is required for efficient translation of viral mRNAs and allows the efficient amplification of Ad, and that VA-RNA II also supports the replication, even though the effect is rather weak compared to that of VA-RNA I. The molecular mechanism of VA-RNAs in supporting the Ad amplification is, at least in part, due to antagonization of the antiviral action associated with the activation of PKR [77]. Following virus infection, dsRNA produced during virus replication can bind to two PKR molecules, leading to autophosphorylation of PKR itself and the subsequent activation of PKR. Activated PKR phosphorylates αsubunit of eukaryotic translation initiation factor-2 (eIF-2) (eIF-2α) [78], leading to the tight association between phosphorylated eIF-2α and eIF-2B [79]. eIF-2B is a guanosine nucleotide exchange factor and plays a crucial role on the conversion of GDP to GTP; however, the association between phosphorylated eIF-2α and eIF-2B captures eIF-2B and prevents the conversion of GDP on non-phosphorylated eIF-2α, resulting in inhibition of the translation of viral mRNA and/or to cell apoptosis [59,78,80-82]. Upon infection with an Ad, VA-RNA I is transcribed from the Ad genome in the infected cells. Subsequently, the VA-RNA I binds to PKR with high affinity, blocks the PKR activation, and enhances viral amplification [75,77,83]. Previous studies using mutant VA-RNAs have demonstrated that the apical stem and the central domain play an essential roles in the association between PKR and VA-RNA I [63,84,85]. In contrast, VA-RNA II only shows limited ability to block the activation of PKR [86]. Furthermore, not all Ad serotypes have VA-RNA II [63]. These suggest that VA-RNA II may not be essential in the Ad amplification.

Recently, several studies have demonstrated that VA-RNAs are also processed in a manner similar to microRNAs (miRNAs) [72,75,87-91]. VA-RNAs are transported to cytoplasm by interaction between those terminal stem and Exportin-5, and are then processed by Dicer. After being processed by Dicer, mivaRNAs are incorporated into the RNA-induced silencing complex (RISC), resulting in production of VA-RNA-derived miRNAs (mivaRNAs). In this processing step, VA-RNAs saturate Exportin-5, Dicer, and RISC, leading to the repression of endogenous miRNA-mediated regulation [88-90]. Surprisingly, approximately 80% of RISC immunopurified from cells transduced with the wild-type Ad is associated with mivaRNAs [89]. Both VA-RNA I and II are processed into mivaRNAs. mivaRNAI-137 and -138 are produced from VA-RNA I (Figure 1); however, only about 5% of total VA-RNA I is processed, because VA-RNAs have larger stem than miRNAs and this processing is not efficient [88-90]. On the other hand, VA-RNA II is mainly processed into mivaRNAII-138 derived from the 3′-strand of VA-RNA II [89,92]. Higher amounts of mivaRNAs derived from VA-RNA II were found in RISC, compared with mivaRNAs derived from VA-RNA I [89]. RISC containing mivaRNAs would suppress expression of target genes, developing cellular environment which is appropriate for virus amplification. Recently, Aparicio *et al.* reported that one of target genes for mivaRNAI-138 is TIA-1, which promotes the expression of proapoptotic proteins [91]. Several genes that are involved in the antiviral action would be suppressed by VA-RNAs in a manner similar to miRNA to support the amplification of Ads.

VA-RNA I is classified into two species, VA-RNA I (A) and VA-RNA I (G), according to the heterogeneity at the 5′-ends derived from the diversity of initiation of transcription [92-94]. VA-RNA I (G) is transcribed 3 nucleotides downstream of the transcription initiation site of VA-RNA I (A), is preferentially synthesized and accounts for about 75% of the total VA-RNA I [92,93]. VA-RNA I (A) and (G) are processed at the same position of the 3′-strand, generating mivaRNAI-137 and 138 (Figure 1) [89]. The 3′strand mivaRNAI is produced at much higher levels compared to the 5′-strand mivaRNAI (A) and (G); however, RISC assembled on the 5′strand mivaRNAI (A) shows higher cleavage activity than that assembled on the 3′strand mivaRNAI [92]. Further examination is required to clarify whether the heterogeneity of the 5′-end of VA-RNA I leads to the differences in mivaRNA-mediated regulation of gene expression.

**Figure 2.**
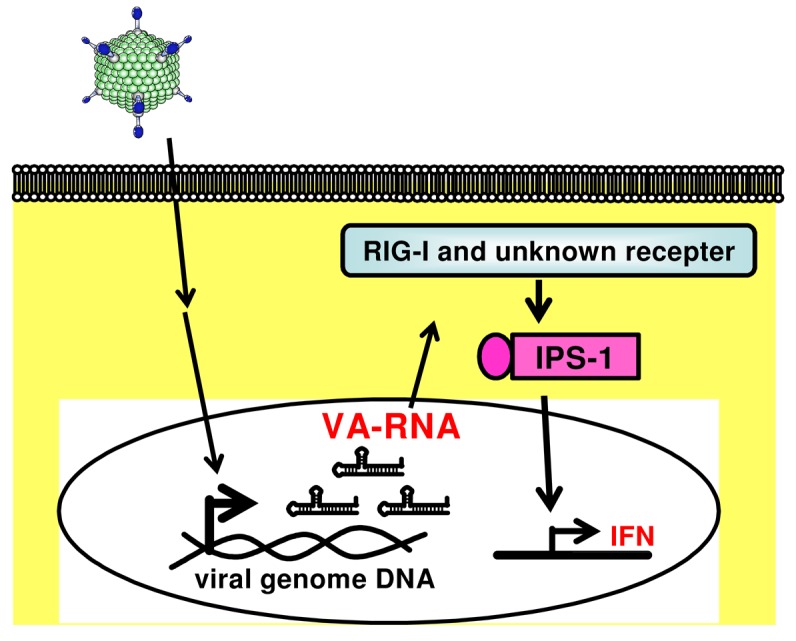
VA-RNA-induced production of type I IFNs through an IPS-1-mediated signaling pathway. VA-RNA: virus-associated RNA; RIG-I: retinoic acid-inducible gene I; IPS-1: IFN-β promoter stimulator-1; IFN: interferon.

Many viruses express some noncoding RNAs which probably control host cellular responses and maximize their replication in the infected host [95,96]. Among the best-characterized viral small RNAs are the Epstein-Barr virus (EBV)-encoded small RNAs (EBERs). EBERs are nonpolyadenylated, untranslated RNAs, transcribed by RNA polymerase III abundantly in latently EBV-infected cells, and form a stem-loop structure. The stem-loop structure of EBERs, which gives rise to dsRNA-like molecules, is recognized by RIG-I and induces expression of type I IFN and IFN-stimulated genes (ISGs) [97]. A recent report by Iwakiri *et al.* demonstrated that EBERs released from EBV-infected cells are recognized by TLR3, and induce type I IFN and proinflammatory cytokines in surrounding noninfected cells as well as in the EBV-infected cells [98]. These evidences suggest that virus-encoded small RNAs are key to not only inducing the innate immune response upon virus infection but also potentially enhancing the following adaptive immunity. We recently demonstrated that VA-RNAs induce the production of type I IFN (IFN-α and IFN-β), but they do not induce the production of inflammatory cytokines (IL-6 and IL-12), in mouse embryonic fibroblasts (MEFs) and granulocyte-macrophage colony-stimulating factor-generated bone marrow-derived dendritic cells (GM-DCs) (Figure 2) [23]. We also showed that IPS-1 is involved in VA-RNA-dependent IFN-β production in MEFs and is partially involved in type I IFN production in GM-DCs (Figure 2) [23]. Moreover, Minamitani *et al.* also reported that VA-RNAs induce the production of type I IFN through an RIG-I pathway [25]. A certain fraction of VA-RNA I possesses triphosphated 5′-termini [94]. Since ssRNA containing 5′-triphosphate activates RIG-I [55,99], 5′-triphosphate of VA-RNA I may be involved in RIG-I-dependent IFN induction. Furthermore, as described above, VA-RNA I is classified into two species, VA-RNA I (A) and VA-RNA I (G) [92-94]. This heterogeneity at 5′-termini of VA-RNA I would lead to the difference in the phosphorylation pattern at 5′-termini. Xu *et al.* demonstrated that higher amounts of mivaRNAI (G) possess the multi-phosphate at 5′-termini, compared with mivaRNAI (A). mivaRNAI (G) might induce type I IFN production more strongly than mivaRNAI (A) in a RIG-I-dependent manner; however, in our analysis using RIG-I-deficient mice, the production of type I IFN is independent of an RIG-I pathway [23].

### 3.2. Development of an Ad vector lacking the expression of VA-RNAs

The findings described above strongly suggest that a VA-RNA-deleted Ad (AdΔVR) vector could be less able to activate the innate immune response, providing a safer alternative to the FG-Ad vectors. Furthermore, development of the AdΔVR vector could provide important insights into unknown functions of VA-RNAs and lead to the discovery of other target genes of mivaRNAs derived from VA-RNAs.

We have developed an AdΔVR vector from which the transcriptional control elements of the VA-RNA expression were deleted. Some previous reports suggested that 293T cells expressing the SV40 large-T antigen or a PKR inhibitor (2-aminopurine, 2-AP) might support the replication and propagation of the AdΔVR vector as follows: the SV40 large-T antigen which antagonizes the translational inhibitory effect resulting from the activation of PKR in SV40-infected cells resulted in counteraction of cellular defense against virus infection [100]; 2-aminopurine (2-AP) interferes with the gene induction normally triggered by IFN, and supports the amplification of a VA-RNA-deleted mutant Ad (Sub720) [78,101]. So far, unfortunately, we have failed to generate an AdΔR vector by these approaches, probably due to the lower capability of propagating Ad vectors in 293T cells and cytotoxicity of 2-AP against HEK293 cells, respectively.

**Figure 3.**
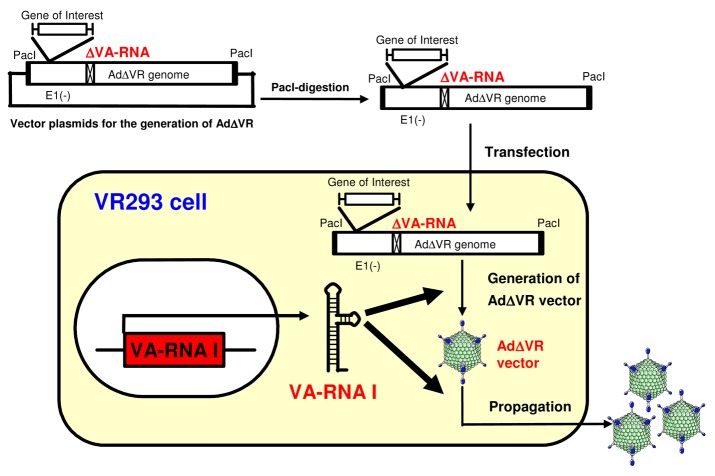
Generation of AdΔVR vectors using VR293 cells. VA-RNA I-expression in VR293 cells allows the generation and the subsequent propagation of VA-RNA-deleted Ad (AdΔVR) vectors.

We prepared VA-RNA I-expressing HEK293 cells (VR293 cells) (Figure 3). VR293 cells allowed the generation and propagation of an AdΔVR vector without the need for any complicated processes or other supporting materials, such as a helper virus, although the recovered titer of the AdΔVR vector was low. The resulting AdΔVR vector showed a high-purity, no expression of VA-RNAs, and an efficient transduction capability [102]. Further studies are needed to develop an improved method for more efficient propagation of AdΔVR vectors (e.g., generation of an improved cell line expressing VA-RNA II as well as VA-RNA I; the use of other PKR inhibitors and IFN-inhibitors with low cytotoxicity). The induction of both the early innate and following adaptive immune responses by the AdΔVR vector should be evaluated *in vitro* and *in vivo* (note that more efficient propagation of AdΔVR vectors is required to analyze the immune response by AdΔVR vectors). The AdΔVR vector could be one of the most variable tools for the analysis of VA-RNA-mediated innate immune response following transduction with Ad vectors.

## 4. Conclusions

In the present paper, we reviewed Ad vector-mediated immunogenic toxicities, especially innate immune responses and VA-RNA. Ad vectors activate innate immune responses through the capsid protein, the viral genome (DNA), and/or viral transcripts. VA-RNAs produced in the Ad vector-transduced cells also stimulate the innate immune responses. It is essential to clarify the mechanism of the innate immune responses triggered by the systemic administration of Ad vectors in order to achieve a safe method of gene therapy using Ad vectors. In addition, such an improved understanding of Ad vector-induced innate immune responses is essential for the successful gene therapy as well as the development of safe vectors.
